# Printing Untethered Self‐Reconfigurable, Self‐Amputating Soft Robots from Recyclable Self‐Healing Fibers

**DOI:** 10.1002/advs.202410167

**Published:** 2024-12-18

**Authors:** Yidan Gao, Wei Tang, Yiding Zhong, Xinyu Guo, Kecheng Qin, Yonghao Wang, Elena Yu. Kramarenko, Jun Zou

**Affiliations:** ^1^ State Key Laboratory of Fluid Power and Mechatronic Systems School of Mechanical Engineering Zhejiang University Hangzhou 310058 China; ^2^ Faculty of Physics Lomonosov Moscow State University Moscow 119991 Russia; ^3^ Enikolopov Institute of Synthetic Polymeric Materials of Russian Academy of Sciences Moscow 117393 Russia

**Keywords:** 3D printing, recyclable self‐healing fibers, self‐amputation, self‐reconfiguration, untethered soft robots

## Abstract

Regarding the challenge of self‐reconfiguration and self‐amputation of soft robots, existing studies mainly focus on modular soft robots and connection methods between modules. Different from these studies, this study focus on the behavior of individual soft robots from a material perspective. Here, a kind of soft fibers, which consist of hot melt adhesive particles, magnetizable microparticles, and ferroferric oxide microparticles embedded in a thermoplastic polyurethane matrix are proposed. The soft fibers can achieve wireless self‐healing and reversible bonding of the fibers by eddy current heating and can be actuated by magnetic fields. Moreover, the soft fibers are recyclable and printable. Building on this material foundation, an integrated material‐structure‐actuation printing strategy using soft fibers for the design and fabrication of soft robots are reported. The robots printed by this strategy can achieve their untethered motions and wireless self‐healing. Soft gripper, soft crawling robot, and soft multi‐legged robot, are then fabricated which demonstrates the self‐healing, self‐reconfigurable, self‐amputating, and sustainable performances of soft robots so as to adapt to different environments and tasks. This integrated material‐structure‐actuation printing strategy using soft fibers is universal, easy to implement, and mass‐manufactured, opening a door for sustainable, eco‐friendly, untethered, self‐reconfigurable, self‐amputating soft robots.

## Graphical abstract

This paper reports an integrated material‐structure‐actuation printing strategy using recyclable and printable soft fibers for the design and fabrication of soft robots. The robots printed by this strategy can achieve untethered motions and wireless self‐healing. A series of soft grippers, soft crawling robots, and soft multi‐legged robots are fabricated by this strategy, which demonstrates self‐reconfigurable, self‐amputating, and sustainable performances.

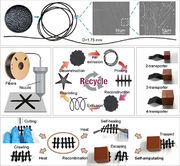

## 1 Introduction

Organisms exhibit extraordinary environmental adaptability through morphological changes.^[^ ^1^ ^]^ Typical morphological change behaviors include self‐reconfiguration^[^ ^2^ ^]^ and self‐amputation.^[^ ^3^ ^]^ Self‐reconfigurable refers to an organism reconfiguring its body structure or a group of organisms reconfiguring their collective arrangement. For example, when an axolotl's limbs are amputated, its body structure can be reconfigured through regeneration;^[^ ^4^ ^]^ and a shoal of fish can accomplish tasks that are challenging for a single individual or original fish school shape by reconfiguring the shape of the fish school.^[^ ^5^ ^]^ Self‐amputating, on the other hand, refers to the behavior of animals amputating appendages to escape danger. For instance, when a gecko's tail is bitten by a predator, it will voluntarily detach its tail to facilitate escape.^[^ ^6^ ^]^ Likewise, when an octopus faces imminent danger, it may sever its own arm to survive.^[^ ^7^ ^]^ These behaviors of living organisms have inspired scientists and engineers to create robots capable of morphological changes.^[^ ^8^, ^9^ ^]^ Robots can adapt to different environments, tasks, or injuries through morphological editing or change, which is one of the frontiers in the field of robotics.^[^ ^10^, ^11^, ^12^ ^]^ Soft robots,^[^ ^13^, ^14^, ^15^, ^16^, ^17^, ^18^ ^]^ with their inherent flexibility and large deformability, have distinct advantages in adapting to unstructured environments.^[^ ^19^ ^]^ Current research on self‐reconfiguration and self‐amputation of soft robots predominantly centers on modular soft robots.^[^ ^20^ ^]^ Such robots employ reversible joints to connect and detach modules,^[^ ^21^, ^22^, ^23^, ^24^ ^]^ allowing for their reconfiguration and amputation. Our previous review papers^[^ ^19^ ^]^ discussed these modular soft robots in detail. Several connection methods^[^ ^19^ ^]^ have been developed for modular soft robots, including mechanical,^[^ ^21^, ^22^, ^25^, ^26^, ^27^, ^28^ ^]^ magnetic,^[^ ^20^, ^23^, ^29^, ^30^ ^]^ adhesive,^[^ ^24^, ^31^, ^32^, ^33^ ^]^ vacuum,^[^ ^34^ ^]^ etc. These studies are mainly aimed at realizing the interconnection and disassembly of modular soft robots. The interconnection and disassembly of modular soft robots^[^ ^20^, ^23^ ^]^ is similar to the swarm behaviors^[^ ^35^, ^36^ ^]^ of insects in nature. In fact, self‐reconfigurable and self‐amputating behaviors of individual organisms^[^ ^2^, ^37^ ^]^ are also very common in nature. Different from existing studies, this study aims to investigate the behavior of individual soft robots from a material perspective, specifically exploring self‐reconfigurable and self‐amputating behaviors of single soft robots or interactions among a few soft robots. We aim to propose an integrated material‐structure‐actuation printing strategy to create soft robots with self‐reconfigurable and self‐amputating behaviors. In addition, due to the increasing threat of robotic waste materials to the environment,^[^ ^38^ ^]^ how to achieve sustainability^[^ ^39^, ^40^, ^41^, ^42^ ^]^ of soft robots from a material perspective is also a focus of this study.

Here, we propose a kind of recyclable self‐healing soft fibers, which consist of hot melt adhesive particles, magnetizable microparticles of neodymium–iron–boron (NdFeB) alloy, and ferroferric oxide (Fe_3_O_4_) microparticles embedded in a thermoplastic polyurethane (TPU) matrix. Hot melt adhesive (HMA) particles can achieve self‐healing and reversible bonding of the fibers through a thermoreversible Diels‐Alder (DA) reaction.^[^ ^43^, ^44^ ^]^ Due to the heating requirement in the DA reaction, we utilize eddy current heating to heat the Fe_3_O_4_ microparticles in the fibers,^[^ ^16^ ^]^ thus enabling wireless self‐healing and reversible bonding of the fibers. NdFeB microparticles impart the fibers with the capability to respond to magnetic fields.^[^ ^45^, ^46^ ^]^ A series of cyclic manufacturing tests demonstrate that self‐healing fibers exhibit good recyclable performance. We use a single‐screw extruder to produce fibers with a diameter of 1.75 mm, making them suitable for desktop fused deposition modeling (FDM) 3D printers. Building upon this material foundation, we report an integrated material‐structure‐actuation printing strategy using recyclable self‐healing fibers for the integrated design and fabrication of soft robots. The benefits of our strategy in integrated rapid prototyping on soft robots are validated using FDM 3D printers. The robots created by this strategy can achieve their untethered motions through magnetic fields and achieve wireless self‐healing after damage through eddy current heating. Soft grippers fabricated by this strategy possess fast grasping ability and good self‐healing performance. By printing multiple soft crawling robots, we showcase the self‐reconfigurable and self‐amputating behaviors of robots. A single soft crawling robot is capable of pushing small objects, while multiple soft crawling robots can reconfigure to form a large soft crawling robot that can push larger objects. When another object outside a small hole needs to be pushed, the large robot can become a small robot through self‐amputating, thus achieving the specified task. After completing their given tasks, the soft gripper and soft crawling robots can be recycled and reprinted into new robots, such as a soft multi‐legged robot, demonstrating that the soft robots produced by our strategy are sustainable and environmentally friendly. The soft multi‐legged robot can cross various complex terrains actuated by a magnetic field, and when some of its legs are pinned down by an object, it can amputate its suppressed legs through eddy currents and then escape danger, just like an octopus amputate its arms when facing danger.

## 2 Results

### 2.1 Design of Soft Fibers

**Figure 1:**
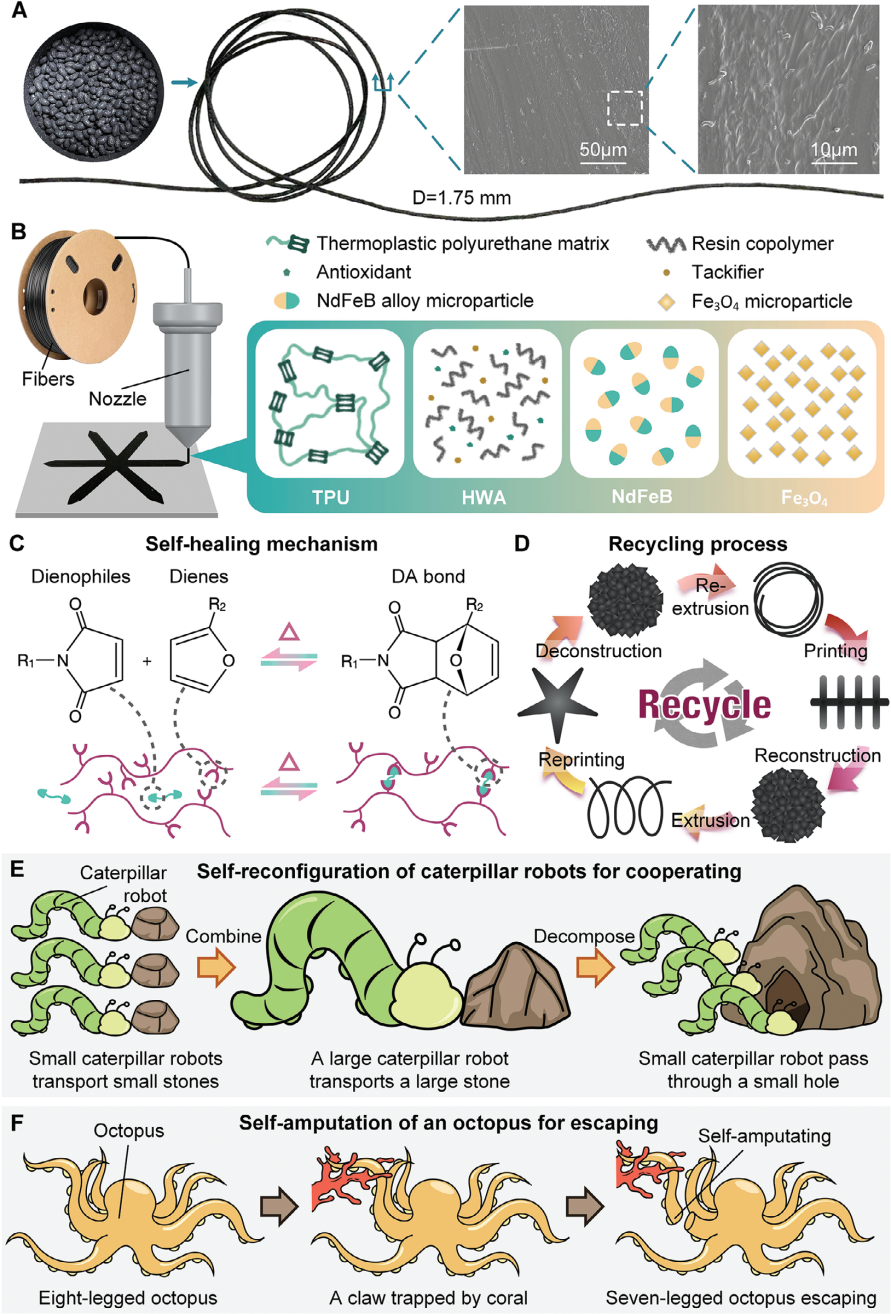
Design of soft fibers. A) Soft fibers fabricated from mixed material particles with a diameter of 1.75 mm and SEM images of the cross‐section surface. B) Schematic diagram of a soft robot printed from soft fibers containing four components (TPU, HWA, NdFeB, and Fe_3_O_4_). C) Schematic diagram of the self‐healing mechanism of soft fibers, molecular diagram, and chain diagram of reversible changes between dienes, dienophiles, and DA bonds. D) Schematic diagram of the recycling process, the conversion cycle among soft fibers, soft robots, and soft fiber fragments. E) The process of self‐reconfiguration of caterpillar robots, combining into a larger caterpillar robot and then decomposing reversely. F) The process of self‐amputation of the octopus, breaking one of the claws and then escaping when trapped by coral.

By fully and uniformly mixing multiple functional material particles including HWA, NdFeB, and Fe_3_O_4_ on the TPU material substrate, the mixed material particles (~3 mm) were melted and extruded to recreate composite soft fibers with a diameter of about 1.75 mm (Movie S1, Supporting Information). The composite soft fibers are shown in **Figure** **1**A and are used to manufacture multifunctional soft robots by 3D printing, and specific manufacturing steps are described in detail in Methods‐Manufacture of soft fibers (Figure S1, Supporting Information). Scanning electron microscopy (SEM) was performed on the cross‐section surface of the composite soft fibers, and the morphology of the mixture was visible in the figure. Among them, TPU is used as the substrate material, accounting for more than 50 wt.%, constituting the matrix phase. HWA forms particles dispersed in a matrix, and these particles are continuously distributed in the matrix. For all the components, it can be observed that the mixed phase has good continuity, and no particles are pulled out of the matrix, indicating a good affinity between all the components.

A soft robot was 3D printed by composite soft fibers using FDM (Movie S2, Supporting Information), and its components are shown in Figure 1B. In contrast to the pour molding manufacturing process of Polydimethylsiloxane (PDMS) and silicone rubber, which is difficult to reprocess,^[^ ^13^, ^14^, ^15^ ^]^ TPU and HWA are used because of their meltable remanufacturing properties. TPU and HWA are easily melted and reconstituted from solid particles at high temperatures and are extruded and cooled into fibers. The other two material particles, NdFeB (38 µm) and Fe_3_O_4_ (10 µm) can be evenly distributed in TPU and HWA through the full melting process to form an integrated material (Figure S2, Supporting Information). The fibers can print any robot by melting and molding again by a 3D printer, because the robot contains multiple functional materials, which can be directly used for actuation after manufacturing, realizing integrated material‐structure‐actuation manufacturing. This soft fiber contains four types of materials, among them, TPU is used as the base material to make the robot soft and stretchable and easy to deform at will. HWA is a self‐healing functional material, the main component is a resin polymer, with the addition of a tackifier for bonding. NdFeB is a magnetic actuation functional material, which will be arranged in a programmed orientation inside the robot after magnetization,^[^ ^45^, ^46^ ^]^ so that the robot will be deformed accordingly under the actuation of the magnetic field, so as to realize the remote untethered actuation of the robot. Fe_3_O_4_ is used in conjunction with energized copper coils to generate an eddy current field that generates heat,^[^ ^16^ ^]^ activating and accelerating the self‐healing process, which is also remotely wireless. Energy dispersive spectroscopy (EDS) of composite soft fibers (Figure S3, Supporting Information) shows that the composite contains B, C, N, O, Fe, and Nd, and according to the distribution and relative content of each element, it can be proved that the composite fiber contains the above four components and does not contain other components.

Figure 1C reveals the mechanism of self‐healing of soft robots, based on the thermally reversible DA response of HWA. The microscopic molecular chain of HWA contains dienes and dienophiles, both of which exist as a DA bond at room temperature.^[^ ^43^, ^44^ ^]^ When the soft robot is injured from the environment, the DA bond at the wound location is mechanically destroyed. When the wound site is heated, and as the heating temperature increases, the DA bonds in the heated position will also decompose into dienes and dienophiles, respectively. As the heating continues, the dienes and dienophiles on the fracture surfaces of the two wounds that are in close contact with each other have sufficient fluidity and are rearranged. After cooling, the fluidity of the dienes and dienophiles gradually decreases until it returns to room temperature, and after enough time, each diene and dienophile on the fracture surface of the two wounds in close contact with each other will combine with each other to form a number of new and stable DA bonds, so that the two surfaces are firmly bonded together and restore their original mechanical properties and functions. During the reaction, the heating temperature does not affect the base TPU material and NdFeB and Fe_3_O_4_ materials, so the form and function of the soft robot are not changed.

To further examine the chemical structure of the composites, Fourier transform infrared spectroscopy (FTIR) analysis was performed (Figure S4, Supporting Information). TPU polymers are composed of hard segments, the hard segments are formed by the reaction of isocyanates with chain extenders, and the soft segments are composed of long polyether or polyester polyol chains. For the TPU figure, the absorption peaks around 2950 and 2860 cm^−1^ are related to the symmetrical and asymmetrical expansion vibrations of the saturated hydrocarbon methyl (─CH_3_) and methylene (─CH_2_─), the peak of 1413 cm^−1^ corresponds to the C═C stretch of the aromatic ring, and the typical absorption peaks of the polyurethane structure exists, including 1722 cm^−1^ (C═O), 1529 cm^−1^ (C─N), and 1076 cm^−1^ (C─O─C), etc. In the TPU‐HWA figure, the absorption peaks of 3324 cm^−1^ and 1528 cm^−1^ are significantly weakened, the peak of 3324 cm^−1^ is attributed to the tensile vibration of N‐H from the hard segment, and the peak of 1528 cm^−1^ is from the in‐plane and out‐of‐plane bending of N─H. In the TPU‐HWA and TPU‐HWA (rDA) figures, the peak at 2995 cm^−1^ is the absorption peak of C─H vibration on the C═C double bond of the six‐membered ring of the DA adduct product, which does not exist in the TPU figure, indicating that the DA bond has been successfully introduced into the composite. Meanwhile, the peak at 2995 cm^−1^ is significantly weakened on the TPU‐HWA (rDA) figure, because at 95 °C, the DA inverse reaction caused the DA bond to break, and the decrease in DA bond content led to the weakening of the absorption peak, which further verifies that this peak was the characteristic peak of the DA adduct product.

TPU and HWA are easily melted from solid particles to form soft fibers and print various soft robots, and fibers or soft robots can also be easily put into re‐melting manufacturing after being chopped into blocks to achieve recycled manufacturing. Moreover, the particle size of NdFeB and Fe_3_O_4_ is micron‐level, and it is always evenly distributed in TPU‐HWA, which does not affect the feasibility of this recycled manufacturing process. Figure 1D illustrates the recycling process of self‐healing fibers and robots. The soft fibers first print a foot‐crawling soft robot, which is shredded into small pieces after completing its task. Then the fibers can be remanufactured from the soft fiber fragments through the extruder machine, completing the recycling of this foot‐crawling soft robot. The recycled fibers can continue to print new robots such as pentagram soft robots according to new needs to achieve new functions. This recycled manufacturing process provides new possibilities for creating sustainable soft robots.

Soft robots can achieve highly flexible self‐reconfiguration and self‐amputating through the self‐healing mechanism. Similar to the mechanism of self‐healing, two objects decompose and recombine DA bonds through heating, and then achieve self‐reconfiguration into one object after cooling. Keeping the DA bonds in a heated decomposition state and physically separating objects can achieve inverse self‐reconfiguration. Similarly, self‐amputating can also be accomplished by heating and breaking down the DA bonds. Figure 1E illustrates the process of self‐reconfiguration of soft robots, where the small caterpillar robots can carry small stones separately, and they can carry a large stone by combining them into a large caterpillar robot. Additionally, the large caterpillar robot can also reversibly self‐reconfiguration and decompose into several small caterpillar robots to pass through small caves. This self‐reconfiguration method is different from the modular robot in that the sub‐robot before the reconfiguration is not a module, but a robot with complete independent complex functions. The integrated material‐structure‐actuation manufacturing enables the self‐reconfiguration process to make multiple independent sub‐robots integrally combine. Figure 1F illustrates the self‐amputation process of a soft robot, in which an eight‐legged octopus escaped by abandoning one of its claws that was trapped in coral and became a free‐roaming seven‐legged octopus. This self‐amputating robot breaks through the limitation of a modular robot that only has a limited number of modules to be connected or disconnected, and it does not have any interfaces but is integrated as a whole which can be truncated or connected at any part of the entire robot.

### 2.2 Printability and Material Behavior of Soft Fibers

**Figure 2:**
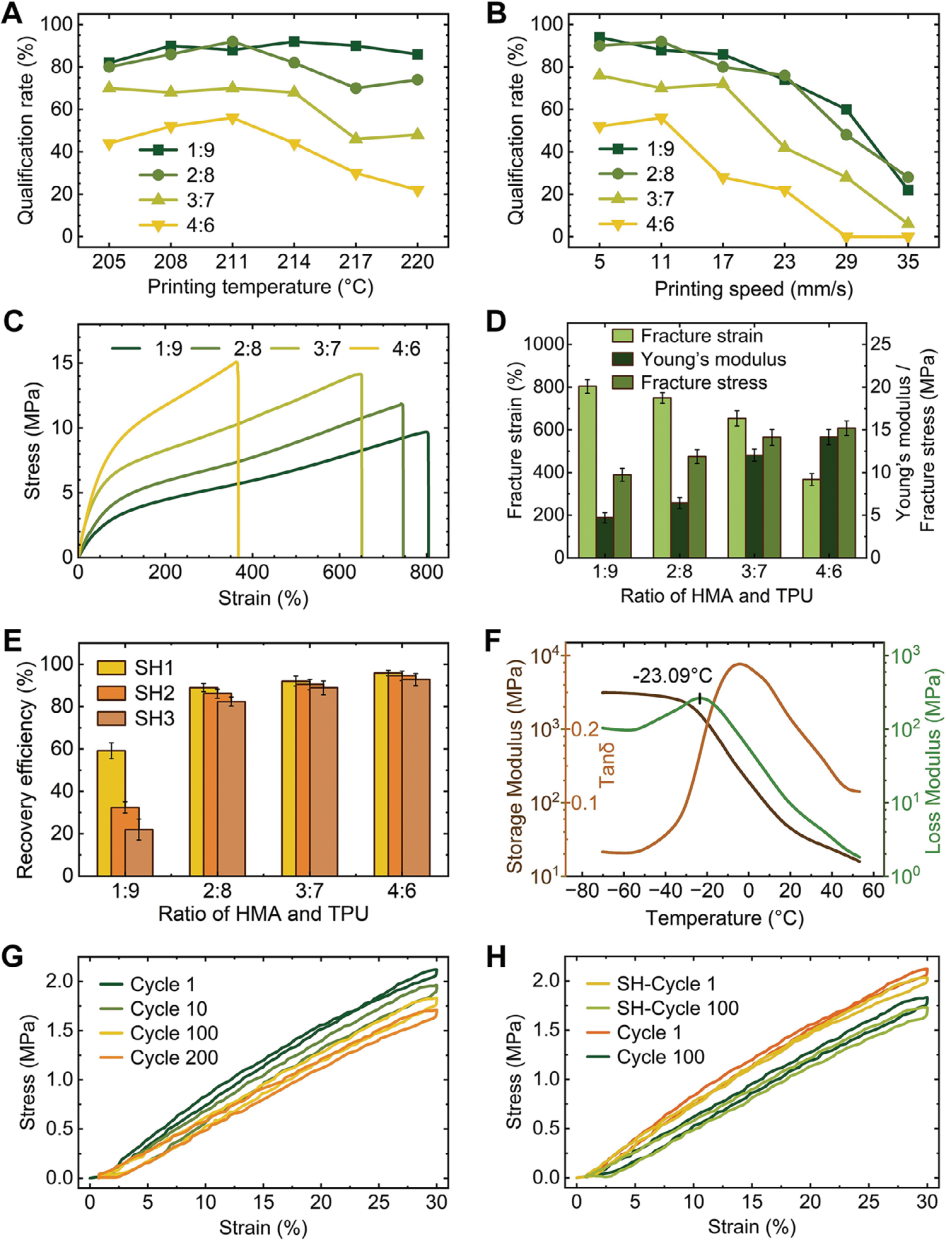
Printability and material behavior of soft fibers. A) For fibers with different composition ratios, the effect of printing temperature on the qualification rate (HMA:TPU = 1:9, 2:8, 3:7, 4:6, the same in B,C,D,E). B) For fibers with different composition ratios, the effect of printing speed on the qualification rate. C) Mechanical properties (stress–strain curves) of fibers with different compositions. D) Young's modulus, fracture stress, and fracture strain values of fibers with different composition ratios. E) The recovery efficiency of fibers with different component ratios after primary self‐healing (SH1), secondary self‐healing (SH2), and tertiary self‐healing (SH3). F) DMA figure of fibers (TPU:HWA:NdFeB:Fe_3_O_4_ is 56:14:10:20), showing their storage modulus, loss modulus, and tanδ. G) Cyclic tensile stress‐strain curves of fibers (TPU:HWA:NdFeB:Fe_3_O_4_ is 56:14:10:20). H, Cyclic tensile stress‐strain curves of fibers before and after self‐healing.

Modifying the printing parameters may result in changes in the properties and functionality of the soft fibers. **Figure** **2**A,B analyze the effects of printing temperature and printing speed on the printing effect of soft fibers with different composition ratios (Methods‐Printing test). The specific operation of the printing test and the definition and calculation method of the qualification rate are explained in detail in the Methods‐Printing test. The printing effect is quantitatively evaluated by the qualification rate. The higher the qualification rate, the better the printing effect, and vice versa. For the printing fiber, keep the proportion of NdFeB and Fe_3_O_4_ particles unchanged at 10 wt.% and 20 wt.%, and adjust the weight ratio of HMA and TPU. According to the figure, it can be concluded that when the ratio of HMA and TPU is 1:9 or 2:8, the printing effect of the fiber is similar at different printing temperatures and printing speeds, while the pass rate of the fiber printing in the ratio of 3:7 and 4:6 is significantly lower than that of the previous ratio. The low printing temperature makes the material difficult to extrude due to insufficient fluidity, while the excessively high printing temperature leads to excessive fluidity of the material, and the print layer is poor and difficult to compact.^[^ ^42^, ^44^ ^]^ Additionally, too high a print speed cannot ensure the time required for good adhesion between the layers, making it difficult for the layers to be deposited uniformly and having poor fixation, while too low a print speed will also lead to too long extrusion time and low reliability of highly viscous materials. Therefore, after testing and research, for the fiber with the HMA: TPU ratio of 2:8, the figure shows that the optimal printing temperature is 208–211 °C, and the optimal printing speed is 5–11 mm s^−1^.

Figure 2C characterizes the mechanical properties of the soft fibers with different composition ratios, and the values of Young's modulus, fracture stress, and fracture strain are shown in Figure 2D. The Young's modulus for HMA and TPU ratio of 2:8 is 6.44 MPa, the fracture strain is 751%, and the fracture stress is 11.87 MPa. In the self‐healing process, the soft robot is heated by an eddy current device (Figure S5, Supporting Information) containing Fe_3_O_4_, so that it can be directly heated and healed in the application scene, without destroying or changing the scene before self‐healing, and can continue to complete its specified action after self‐healing. Moreover, the eddy current device has a relatively small heating area, which can realize point‐to‐point accurate heating and avoid DA inverse reaction in other parts of the robot. Samples were tested for self‐healing performance (Figure S6, Supporting Information and Methods‐Self‐healing test), being heated at 80 °C for 6 h and then cooled at room temperature (25 °C) for 6 h. The specific operation of the self‐healing test and the definition and calculation method of recovery efficiency are explained in detail in the Methods‐Self‐healing test. The self‐healing ability is quantitatively evaluated by the recovery efficiency. The higher the recovery efficiency, the better the self‐healing ability, and vice versa. The recovery efficiency is shown in Figure 2E, it can be seen that the recovery efficiency of the fiber with the ratio of HMA and TPU of 1:9 is obviously low, and the primary recovery efficiency of the 2:8 ratio of the fiber reaches 88%, while the primary recovery efficiency of the 3:7 or higher ratio of the fiber reaches more than 90%. When the ratio of HMA and TPU of fiber is 2:8 or higher, it can be seen that there is only a small decrease in the secondary and tertiary recovery efficiency of fibers. Considering the above factors, the material component ratio of the fiber manufactured in the following studies is set to be 56:14:10:20 as TPU:HWA:NdFeB:Fe_3_O_4_, the printing temperature is set to be 210 °C, and the printing speed is 8 mm s^−1^.

Figure 2F performs dynamic mechanical analysis (DMA) to characterize the viscoelastic properties of the sample, which shows that the glass transition temperature (T_g_) of the composite is −23.09 °C. When T > T_g_, the movement of the molecular chain is no longer restricted, and the storage modulus decreases. The storage modulus of the composite material at room temperature (25 °C) is 36.36 MPa, which is due to the high modulus of TPU itself and the good dispersion of the composite material in the TPU substrate, but it still shows the characteristics of flexible polymer. The loss modulus is 7.12 MPa, which indicates that the viscosity of the composite material is good. The tanδ is 0.196, indicating the elastic behavior of the material, which can be used for printing and manufacturing soft robots.

In order to verify the elastic properties and fatigue resistance of the prepared composite material, durability tests were conducted. 33.3% of the maximum elastic strain (about 90% strain) was selected to obtain its cyclic tensile stress–strain curve, and 200 low‐frequency loading‐unloading cycles were measured. As shown in Figure 2G, after 200 stretches, the material can still recover its original shape with a maximum stress loss of 18.8% and only slight plastic deformation, proving its good elastic properties and durability. In addition, Figure 2H compares the elastic durability of the fibers before and after self‐healing. The maximum stress loss of the composite material after 100 cycles is 12.7%, while the maximum stress loss of the self‐healing fiber after 100 cycles is 13.6%, indicating that this composite material still maintains its elastic properties after self‐healing.

### 2.3 Characterization of Self‐Healing, Actuation and Recyclability

**Figure 3:**
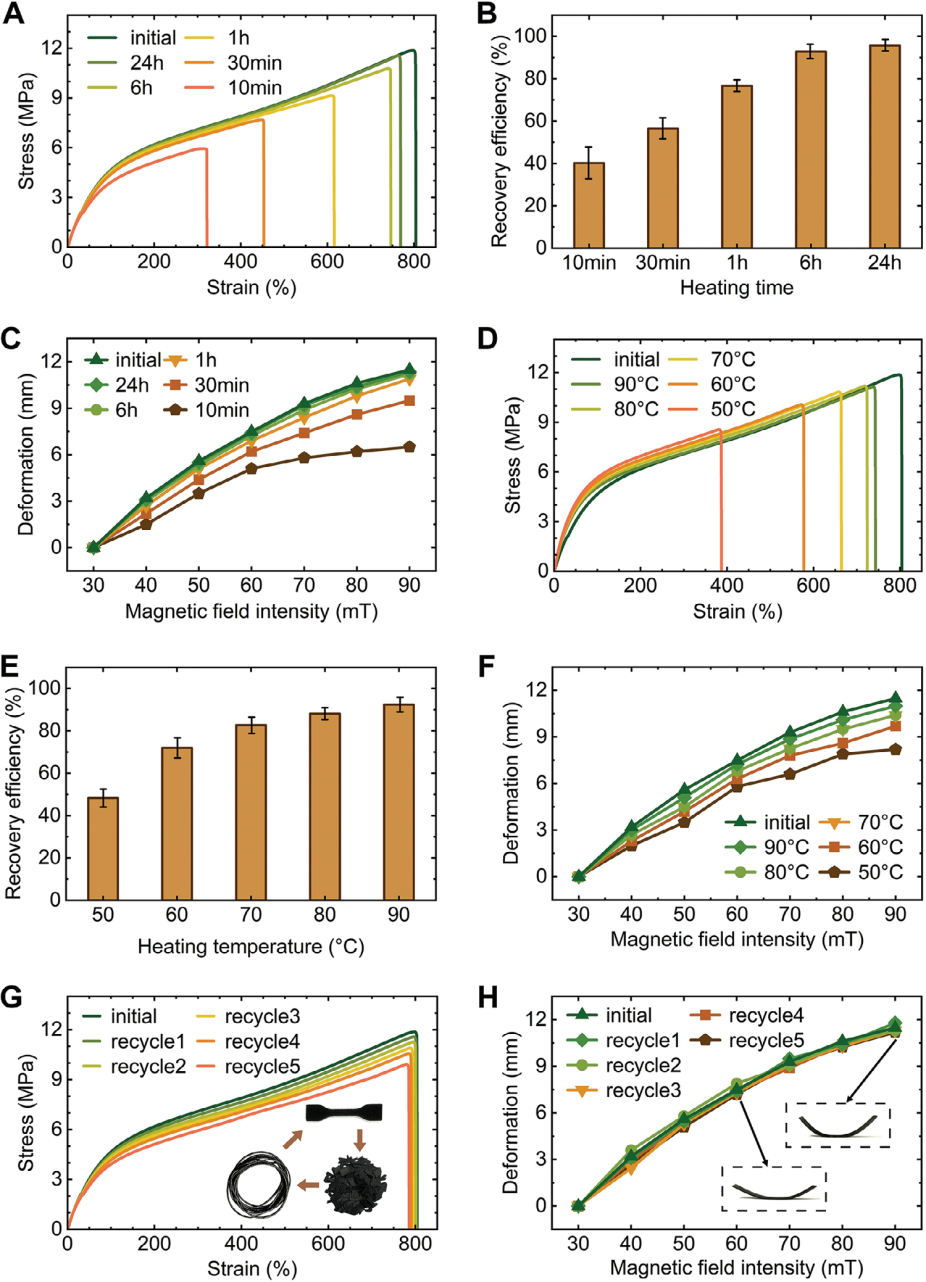
Characterization of self‐healing, actuation, and recyclability. A) Mechanical properties (stress–strain curves) of soft fibers after self‐healing at 90 °C with different heating times. B) Recovery efficiency of the fibers after self‐healing at 90 °C after different heating times. C) Actuation performance of soft fibers after self‐healing at 90 °C with different heating times. D) Mechanical properties (stress–strain curves) of soft fibers after self‐healing after heating for 6 h at different heating temperatures. E) Recovery efficiency of the fiber after heating for 6 h at different heating temperatures. F) Actuation performance of soft fibers after heating for 6 h at different heating temperatures for self‐healing. G) Mechanical properties (stress–strain curves) of soft fibers after multiple cycles. H) Actuation performance of soft fibers after multiple cycles.

In order to study the effect of heating time on the self‐healing properties of the soft fibers, multiple sets of samples were printed self‐healing tests were carried out, and the heating temperature was set to 90 °C. **Figure** **3**A shows the mechanical properties of the fibers after self‐healing at 90 °C with different heating times, and the fracture stress and fracture strain increase with the increase of heating time. Figure 3B shows the recovery efficiency of the fiber at 90 °C with different heating times, and its recovery efficiency increases with the heating time, reaching 93% after 6 h of heating and 96% after 24 h of heating.

The soft robot is controlled by the magnetic field, and a variety of soft robots are designed according to different requirements, and the specified motions can be directly actuated by the magnetic field after 3D printing, which demonstrates the integrated material‐structure‐actuation manufacturing strategy, and the magnetic field is controlled remotely to avoid direct contact. In order to study the effect of heating time on the self‐healing magnetic actuation performance of materials, multiple sets of samples were printed, and self‐healing magnetic actuation tests were carried out on the samples (Figure S7, Supporting Information and Methods‐Magnetic actuation test), defining the amount of deformation (Figure S8, Supporting Information) to reflect the quality of the actuation performance. Figure 3C shows the actuation performance of a sample of this fiber after self‐healing at 90 °C with different heating times, and after heating for 1 h or more, the sample shows an actuation performance close to that of the original sample.

In order to study the effect of heating temperature on the self‐healing properties of the material, multiple sets of samples were printed self‐healing tests were carried out, and the heating time was set to 6 h. Figure 3D shows the mechanical properties of the samples after self‐healing after heating for 6 h at different heating temperatures, the fracture stress and fracture strain increase with the increase of heating time. Figure 3E shows that the recovery efficiency of the samples after heating for 6 h at different heating temperatures increases with the heating temperature, and the recovery efficiency can exceed 80% at 70 °C, while the recovery efficiency reaches 89% at 80 °C, and 92% at 90 °C. In order to study the effect of heating temperature on the self‐healing magnetic actuation performance of the fibers, multiple sets of samples were printed and the self‐healing magnetic actuation test was carried out. Figure 3F shows the actuation performance of the sample after heating for 6 h at different heating temperatures. The magnetic actuation performance of the sample after healing gradually improves with the increase of heating temperature, and the self‐healing sample at 80 and 90 °C heating temperature basically recovers the original actuation performance. In order to study the effect of recycling on the mechanical properties and actuation properties of the fibers, multiple sets of samples were printed and cyclic tests were carried out. Figure 3G,H show the mechanical properties and actuation properties of the fibers after multiple shredding, extruding, and printing cycles. It can be seen that both the strain‐stress curve and the actuation deformation curve change slightly, indicating the good recyclability of the soft fibers.

### 2.4 Self‐Healing Soft Gripper

**Figure 4:**
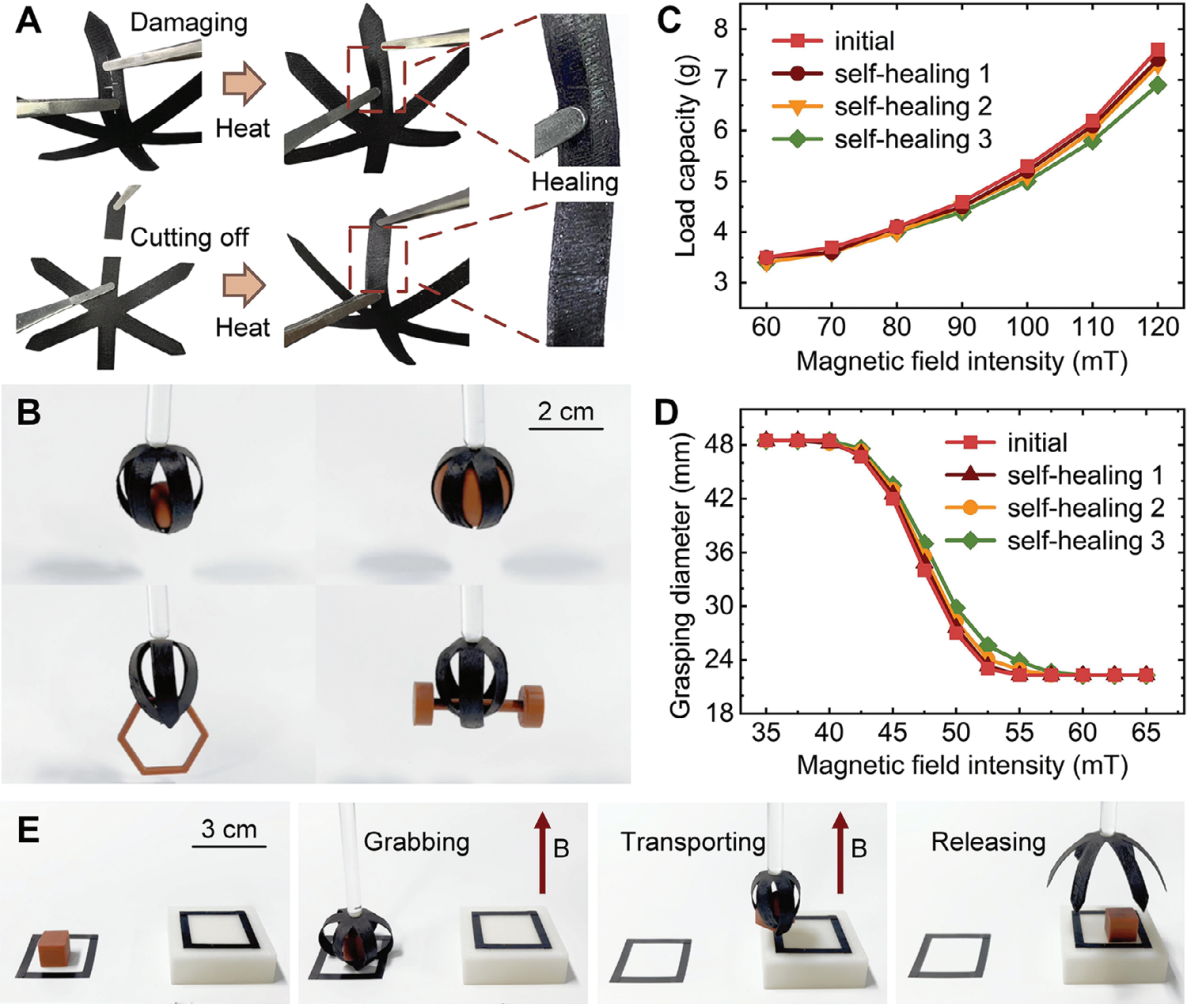
Self‐healing soft gripper. A) A cross damage in the soft gripper was cut by a knife, also one of the claws was cut off, and both these damages have been heated to self‐healing. B) The self‐healing soft gripper which recovers from cutting is able to grasp objects of different sizes and shapes. C) The original load capacity of the soft gripper and after the primary, secondary, and tertiary self‐healing is compared, by testing the maximum weight the soft gripper can hold under different magnetic fields. D) The size of the grasping space of the original soft gripper and after the primary, secondary, and tertiary self‐healing is compared, by testing its maximum diameter when actuated by a magnetic field. E) The self‐healing soft gripper is demonstrated to grasp and transfer an object to the corresponding area in the high position.

A six‐claw soft gripper was printed using the soft fibers fabricated above, which were used to grasp various objects. The fiber imparts self‐healing properties to the soft gripper. **Figure** **4**A shows that the soft gripper was cut by a knife, resulting in cross damage that had to be healed by heating. In addition, when one of the claws of the soft gripper is cut off, the severed claw is fixed in place and heated. After cooling, the soft gripper returns to its original form, and the severed claw is not deformed under the external force stretching (Movie S3, Supporting Information). Figure 4B shows that the self‐healing soft gripper which recovers from cutting not only regains its form, but also regains its original function, capable of grasping objects of different sizes and shapes, such as balls of different diameters, hexagonal frames, and dumbbell‐shaped objects (Movie S4, Supporting Information).

In order to study the soft gripper's repeated self‐healing performance, a second cut was made at the same location as the first one, and similarly the third one. Figure 4C evaluates the load capacity of the soft gripper after the primary, secondary, and tertiary self‐healing. The load capacity of the soft gripper increases with the increase of the actuation magnetic field strength, and the soft gripper can hold a load more than 10 times its own weight (0.62 g) in an actuation magnetic field of 120 mT. After several self‐healing cycles, the soft gripper's load capacity was almost unaffected. Only under strong magnetic fields, the load capacities of the tertiary self‐healing soft grippers were considerably reduced. The grasping space size after primary, secondary, and tertiary self‐healing is compared with the original gripper in Figure 4D. The gripper can be actuated under a magnetic field stronger than 40 mT, as the magnetic field strength increases, the diameter of the grasping space decreases until the soft gripper reaches the minimum grasping space diameter under the magnetic field stronger than 55 mT, that is, the normal grasping state. There is no obvious change in the size of the grasping space of the soft gripper after repeated self‐healing, but the more repeated self‐healing times, the smaller the shrinkage of the grasping space, which causes the tertiary self‐healing soft gripper to reach the normal grasping state under the magnetic field stronger than 60 mT. Figure 4E demonstrates the function of the self‐healing soft gripper to grasp and transfer the object. When a vertical upward 120 mT magnetic field is applied, the soft gripper grabs the object and then transfers it. After removing the magnetic field, the soft gripper releases the object, and the object is finally transported to the corresponding area in a high position (Movie S5, Supporting Information).

### 2.5 Reversible Self‐Reconfigurable Soft Crawling Robot

**Figure 5:**
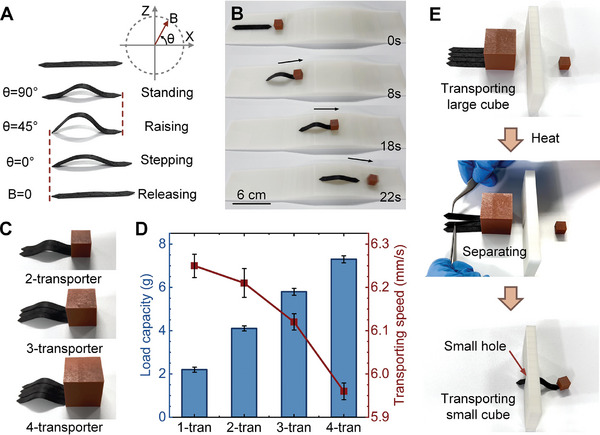
Reversible self‐reconfigurable soft crawling robot. A) The soft crawling robot alternates between standing, raising, stepping, and releasing motion under the rotating magnetic field with different magnetic field angles. B) The soft crawling soft robot transports the object across the bridge. C) 2–4 identical soft crawling robots form large robots and correspondingly transport larger objects. D) The load capacity and transporting speed of different sizes of soft crawling robots. E) The reversible self‐reconfiguration process of soft crawling robots, the large robot composed of four sub‐robots can also decompose into four small robots to complete different tasks.

A soft crawling robot was printed using the self‐healing soft fibers. As **Figure** **5**A shows, the robot stands up when applying a vertical upward magnetic field, and then lifts its tail as the magnetic field is rotating clockwise. Until the magnetic field continuously rotates in a parallel direction, the robot's head takes a step forward, and the robot returns to a relaxing state after removing the magnetic field. This motion cycle of the soft crawling robot is cycled and the rotating magnetic field actuates the robot crawling forward. This robot is capable of transporting objects on flat ground and slopes, such as Figure 5B shows that the robot is able to smoothly carry a cube object across the bridge (Movie S6, Supporting Information).

The robot's transport capacity is limited in terms of the weight and size of the object. Larger objects are expected to be carried by two identical robots, but their weak movement when actuated by a magnetic field makes the task challenging to do. Therefore, several identical robots can be combined into a larger and more powerful robot by self‐healing, which achieves self‐reconfiguration and can be used to transport larger objects. As shown in Figure 5C, based on the self‐reconfigurable property, i soft crawling robots become an i‐transporter (i‐tran, i = 1, 2, 3, 4) by heating. These transporters crawl as the single soft crawling robot does, in order to transport larger objects (Movie S7, Supporting Information). Figure 5D shows the load capacity and transporting speed of different sizes of transporting robots, with a nearly proportional increase in load capacity and a slight decrease in the transporting speed as the number of combined robots increases. Figure 5E demonstrates the reversible self‐reconfiguration process of the soft crawling robot. A large crawling robot (4‐tran) composed of four sub‐robots moves a large cube to a specified location and meets an impenetrable wall. Then the robot is heated by eddy current with a portable coil to remove the position restriction between the sub‐robots, and the large robot breaks down into four robots (1‐tran), which go through a small hole in the wall and move the small cube in the other side (Movie S8, Supporting Information).

### 2.6 Sustainable, Self‐Amputating Soft Multi‐Legged Robot

**Figure 6:**
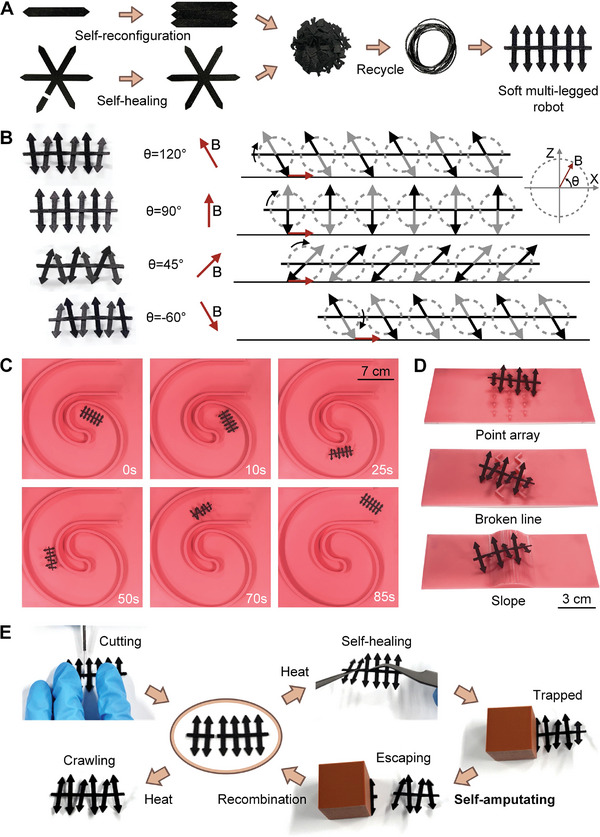
Sustainable, self‐amputating soft multi‐legged robot. A) A soft multi‐legged robot is printed through recycling from the previous soft grippers and soft crawling robots. B) The locomotion mechanism of the soft multi‐legged robot under a rotating magnetic field. The schematic diagram is in the side view, with black feet representing the foot closer to the view and gray feet representing the foot farther away from the view. C) The process of the soft multi‐legged robot crawling out of the maze of circles. D) The forms of the soft multi‐legged robot to traverse complex terrains such as point arrays, broken lines, and slopes. E) Self‐amputating and self‐reconfiguration process of a soft multi‐legged robot.

As **Figure** **6**A shows, a soft multi‐legged robot is printed through recycling. The self‐healing soft grippers and the self‐reconfigurable soft crawling robots are cut into small pieces and added to the extruder machine to manufacture recycling soft fibers. The components and properties of the recycled soft fibers are the same as the origin. The printed soft multi‐legged robot also has the capabilities of eddy current heating, self‐healing, and magnetic actuation, which confirms that the soft robot produced by our strategy is sustainable and environmentally friendly. Figure 6B shows the locomotion mechanism of the soft multi‐legged robot under a rotating magnetic field. The six pairs of legs on the robot are actuated by a rotating magnetic field that rotates in the X‐Z plane at a constant speed. The adjacent two pairs of feet are actuated toward opposite directions and rotate clockwise with the magnetic field synchronously, while the angle between the feet and the ground is the same as the magnetic field direction. Based on this locomotion mechanism, the multi‐legged robot's feet on either side of the body touch the ground alternately, and the frictional force between its feet and the ground propels the robot to move forward. By adjusting the angle between the plane of the rotating magnetic field and the X‐Z plane at a constant speed, the soft multi‐legged robot can be actuated to make a turn, and Figure 6C shows the soft multi‐legged robot crawling out of the maze of circles (Movie S9, Supporting Information). The soft crawling robot can adapt to complex and uneven terrains due to its multiple pairs of feet, Figure 6D shows that the soft multi‐legged robot stably traverses terrains such as point arrays, broken lines, and slopes (Movie S10, Supporting Information), and the feet of different heights can adapt to uneven ground.

Figure 6E demonstrates the self‐amputating process of the soft multi‐legged robot. In the beginning, the robot's body is cut off by a knife, and by fixing its body to heat, the robot has been successfully self‐healing and crawls again after cooling. Then self‐healing robot is unexpectedly trapped by a heavy object during crawling and it is impossible to break free. In this case, the portable coil is used to heat the pinned part of the robot with eddy current, and the trapped two pairs of feet are immediately disconnected, which enables the soft robot to escape with the remaining four pairs of feet actuated by the magnetic field. After that, the quadruped robot and the eight‐legged robot are recombined and heated, which self‐reconfigurate into the original multi‐legged robot and resumes its original crawling function under the actuation of the magnetic field (Movie S11, Supporting Information).

## 3 Discussion

In this study, a new kind of composite multifunctional self‐healing soft fibers was fabricated, an integrated material‐structure‐actuation printing strategy was proposed, and multiple soft robots with self‐reconfigurability, self‐amputation, and sustainability were designed and manufactured on this basis. We proposed a soft fiber with TPU as the substrate material doped with functional materials such as hot melt adhesive particles, NdFeB particles, and Fe_3_O_4_ particles, and fabricated the soft fibers with an extruder. By 3D printing, we used the soft fibers to create soft grippers, soft crawling robots, and soft multi‐legged robots. These soft robots are capable of deformation and motion under the control of magnetic fields, self‐healing under eddy current heating, and complete functions and behaviors related to self‐reconfiguration, self‐amputating, and recycling.

Compared with the manufacturing process of silicone rubber and PDMS,^[^ ^13^, ^14^, ^15^ ^]^ our study uses melt‐reconstituted TPU and hot melt adhesive granular materials. This advantage makes it easy for our materials to be formed into fibers by the extruder for 3D printing, providing the material basis for an integrated material‐structure‐actuation printing strategy. In addition, this property also makes our fibers and soft robots recyclable, contributing to environmental protection and sustainable development.^[^ ^39^, ^40^, ^41^, ^42^ ^]^ Compared with the existing research on modular soft robots, their functions are mainly completed by the connection between modules,^[^ ^19^, ^20^, ^21^, ^22^, ^23^, ^24^ ^]^ but our study aims to develop the functions of individual soft robots. Soft robots are fully functional integrated individual robots or robot groups before and after self‐reconfiguration and self‐amputation, which adapt to different tasks and environments through self‐healing mechanisms, emphasizing the integration of material‐structure‐actuation of soft robots. Compared with the traditional control that may have an impact on the robot itself,^[^ ^25^, ^43^ ^]^ our study uses wireless remote control in both self‐healing and actuation. Soft robots made of soft fibers can achieve wireless self‐healing through eddy current heating, so as to complete self‐reconfiguration and self‐amputation, and are remotely actuated by a magnetic field to complete the corresponding motions. Soft robots printed through this strategy can achieve wireless self‐healing and untethered motion, which is also superior in terms of actuation.

Our integrated material‐structure‐actuation printing strategy uses soft fibers to fabricate untethered, sustainable, self‐reconfigurable, and self‐amputating soft robots. This strategy is versatile, easy to implement, and mass‐manufactured, and is expected to be promoted to flexible electronics, wearable devices, and biomedicine.

## 4 Experimental Section

### Materials

TPU Granules (90A, Dongguan Guangyuan Plasticizing Co., Ltd., China), HWA Granules (70A, Foshan Gaffis Plastic Products Co., Ltd., China), NdFeB Powder (LW‐N, Guangzhou Xinnuode Transmission Components Co., Ltd., China), Fe_3_O_4_ Powder (≥97%, I811694, Shanghai McLean Biochemical Co., Ltd., China)

### Manufacture of Soft Fibers

The TPU pellets were first dried in a vacuum oven at 80 °C for 4 h. NdFeB powder and Fe_3_O_4_ powder were added proportionally and prepared in a heated mixer at 175 °C at 60 rpm for 5 min. After mixing well, HMA particles were added in proportion at a temperature of 30 °C and a speed of 60 rpm for 5 min, and finally, evenly distributed mixed material particles were obtained.

The mixed material particles were fed into the hopper of the single‐screw extruder (Wellzoom C model) and were pushed forward under the rotation of the screw at 7.2 rpm. The particles gradually melted under heating at 200 °C in the mixing section and were finally extruded at 205 °C in the extrusion section. After being extruded, the fibers were pulled by a tractor and cooled to room temperature through a water cooling process. Adjusting the speed of the tractor could adjust the fiber diameter before the fiber completely cools down, and finally, smooth fibers with a diameter of about 1.75 mm were formed. The fibers were rolled into a disc of 3D‐printed filaments by a rolling machine, ready to be used to manufacture soft robots.

This manufacturing method could produce a large amount of fibers for commercial 3D printing filaments, which could be widely printed using commercial 3D printers. In this experiment, the printer used for printing is the model Ender‐3 S1 produced by Creality.

### Measurement and Characterization

Scanning electron microscopy (SEM) was obtained by Quattro S from Thermo Fisher. Energy dispersive spectroscopy (EDS) was obtained by Octane Elect Plus from EDAX. Fourier transform infrared spectroscopy (FTIR) was tested on a Nicolet 380 spectrometer in attenuated total reflectance mode (ATR) with spectral sweep wavenumbers ranging from 600 to 4000 cm^−1^, 32 scans, and a resolution of 4 cm^−1^. TPU and TPU‐HMA samples were tested respectively at room temperature (25 °C), and then the TPU‐HMA sample was heated at 95 °C for 1 h, and test the TPU‐HMA sample again. The tensile test was performed using a dumbbell‐shaped sample (50 × 4 × 1 mm^3^) on an INSTRON model 34SC‐05 tensile testing machine with a tensile rate of 100 mm min^−1^ and an initial spacing between grippers of 20 mm. Dynamic mechanical analysis (DMA) was performed using the Q4.5 from Universal V800A TA Instruments, using 0.1% oscillatory strain and a frequency of 1Hz, force tracking of 125%, and heating of 10 K min^−1^.

### Magnetization of Soft Robots

According to the pre‐programmed form of the soft robot to be magnetized, the hard mold was printed to fix the form and position of the soft robot. The mold and the soft robot were placed vertically in a box wrapped around the coil, and the magnetizer (MAT‐3030 JIUJUOK) instantly applied a voltage of about 3000 V to generate a pulse field of about 1.8 T to magnetize the soft robot. Before the soft robot was shredded and recycled, it was demagnetized using the same equipment.

### Actuation for Soft Robots

The magnetic field generator consisted of three pairs of Helmholtz coils perpendicular to each other, controlled by a magnetic field control system, which could generate a magnetic field (0–120 mT) in space. The soft robot was placed in the center of the magnetic field generator, and the size and direction of the magnetic field can be adjusted in the X, Y, and Z directions through the PS2 wireless handle, which was used to remotely actuate the magnetic soft robot.

### Printing Test

The layer height was set to 0.2 mm, the trace width to 0.4 mm, the fill rate to 110%, the print platform temperature to 40 °C, and the printing speed and printing temperature required for the test were set. For each speed and temperature condition, 5 samples (50 × 10 × 1 mm^3^) were printed at a time for a total of 10 times. The entire printing process was monitored, and if each layer and each line of the sample was printed completely, it was called a qualified sample. The qualification rate of different test conditions was obtained by the following formula. Of these, the total number of samples is 50.

(1) $$\mathrm{Qualification}\;\mathrm{rate}=\frac{\mathrm{Number}\;\mathrm{of}\;\mathrm{qualified}\;\mathrm{samples}}{\mathrm{Total}\;\mathrm{number}\;\mathrm{of}\;\mathrm{samples}}\times 100\%$$

### Self‐Healing Test

A knife to cut the middle of the sample (50 × 10 × 1 mm^3^) obliquely was used, the incision was tightly compact, the eddy current device was turned on, the center of the copper coil was placed directly above the incision, the heating time and heating temperature was set, the distance between the copper coil and the sample wa adjusted to control the heating temperature (displayed on the display), then the eddy current device (or use an oven instead) was removed, and cooled for 4 h. For the same dumbbell‐shaped sample (50 × 4 × 1 mm^3^), the self‐healing operation under different conditions should be first performed, and then the tensile test should be performed on each sample. The recovery efficiency of different test conditions was obtained by the following formula.

(2) $$\mathrm{Recovery}\;\mathrm{efficiency}=\frac{\mathrm{Current}\;\mathrm{fracture}\;\mathrm{strain}}{\mathrm{Initial}\;\mathrm{fracture}\;\mathrm{strain}}\times 100\%$$

To perform a repeated self‐healing test (SH2 and SH3), the sample again was cut at the first position of the incision healing and repeat the self‐healing procedure.

In the self‐healing magnetic actuation test, the sample (50 × 10 × 0.3 mm^3^) that had already been magnetized should go through the self‐healing procedure first, and then perform the magnetic actuation operation.

### Magnetic Actuation Test

The sample (50 × 10 × 0.3 mm^3^) was fixed into a preset U‐shape with a mold, magnetized, and then placed in the magnetic field control device, the magnetic field strength was set, and the vertical upward magnetic field was applied, and the deformation d of the sample was measured. For the self‐healing magnetic actuation test, self‐healing should be done between magnetization and magnetic actuation steps.

## Conflict of Interest

The authors declare no conflict of interest.

## Author Contributions

Y.G. and W.T. contributed equally to this work. W.T., J.Z., and E.Y.K. proposed and supervised the project. W.T., J.Z., and Y.G. designed the research. Y.G., Y.Z., X.G., K.Q., and Y.W. conducted the experiments. Y.G. and W.T. analyzed the data. Y.G. and W.T. wrote the manuscript. W.T., E.Y.K., and J.Z. revised the manuscript. All authors participated in discussions of the research and revisions of the manuscript.

## Supporting information



Supporting Information

Supplemental Movie 1

Supplemental Movie 2

Supplemental Movie 3

Supplemental Movie 4

Supplemental Movie 5

Supplemental Movie 6

Supplemental Movie 7

Supplemental Movie 8

Supplemental Movie 9

Supplemental Movie 10

Supplemental Movie 11

Supplemental Movie 12

## Data Availability

The data that support the findings of this study are available in the supplementary material of this article.
