# Supplementary material for: Printing Untethered Self‐Reconfigurable, Self‐Amputating Soft Robots from Recyclable Self‐Healing Fibers
Source: Adv Sci (Weinh). 2024 Dec 18;12(6):2410167. doi: 10.1002/advs.202410167 (PMC11809436; doi:10.1002/advs.202410167)
Supplement: Supplementary file 1 — Supporting Information [file ADVS-12-2410167-s005.docx]

**Supplementary Materials**

**Printing untethered self-reconfigurable, self-amputating soft robots from recyclable self-healing fibers**

Yidan Gao^1#^, Wei Tang^1#^*, Yiding Zhong^1^, Xinyu Guo^1^, Kecheng Qin^1^, Yonghao Wang^1^, Elena Yu. Kramarenko^2,3^*, Jun Zou^1^*

^1^State Key Laboratory of Fluid Power and Mechatronic Systems, School of Mechanical Engineering, Zhejiang University, Hangzhou 310058, China

^2^Faculty of Physics, Lomonosov Moscow State University, Moscow 119991, Russia

^3^Enikolopov Institute of Synthetic Polymeric Materials of Russian Academy of Sciences, Moscow 117393, Russia

*Corresponding author. Email: junzou@zju.edu.cn (Jun Zou), kram@polly.phys.msu.ru (Elena Yu. Kramarenko), weitang@zju.edu.cn (Wei Tang)

^#^These authors contributed equally to this work

**This PDF file includes:**

Fig. S1. Fabrication processes of soft fibers and soft robots.

Fig. S2. SEM images of the printed sample surface.

Fig. S3. Energy dispersive spectroscopy (EDS) image.

Fig. S4. Fourier transform infrared spectroscopy (FTIR) - attenuated total reflection mode (ATR) image.

Fig. S5. Diagram of an eddy current heating device.

Fig. S6. Schematic diagram of self-healing performance test.

Fig. S7. Schematic diagram of magnetic actuation performance test.

Fig. S8. Deformation of the magnetic actuation test.

Fig. S9. Dimensions and magnetic pre-programming status of soft robots.

Movie 1. Fabrication process of soft fibers with an extruder.

Movie 2. 3D printing of soft robots using soft fibers.

Movie 3. Repeated self-healing process of a soft gripper.

Movie 4. Demonstration of a self-healing soft gripper grasping different objects.

Movie 5. Transferring process of an object with a self-healing soft gripper.

Movie 6. Demonstration of a soft crawling robot transporting an object across a bridge.

Movie 7. Self-reconfiguration process of soft crawling robots.

Movie 8. The reversible self-reconfiguration process of soft crawling robots.

Movie 9. Demonstration of a soft multi-legged robot crawling out of a maze.

Movie 10. Demonstration of a soft multi-legged robots traversing complex terrains.

Movie 11. Self-amputation escaping process of a soft multi-legged robot.

Movie 12. Locomotion of a soft multi-legged robot with and without feet.

**
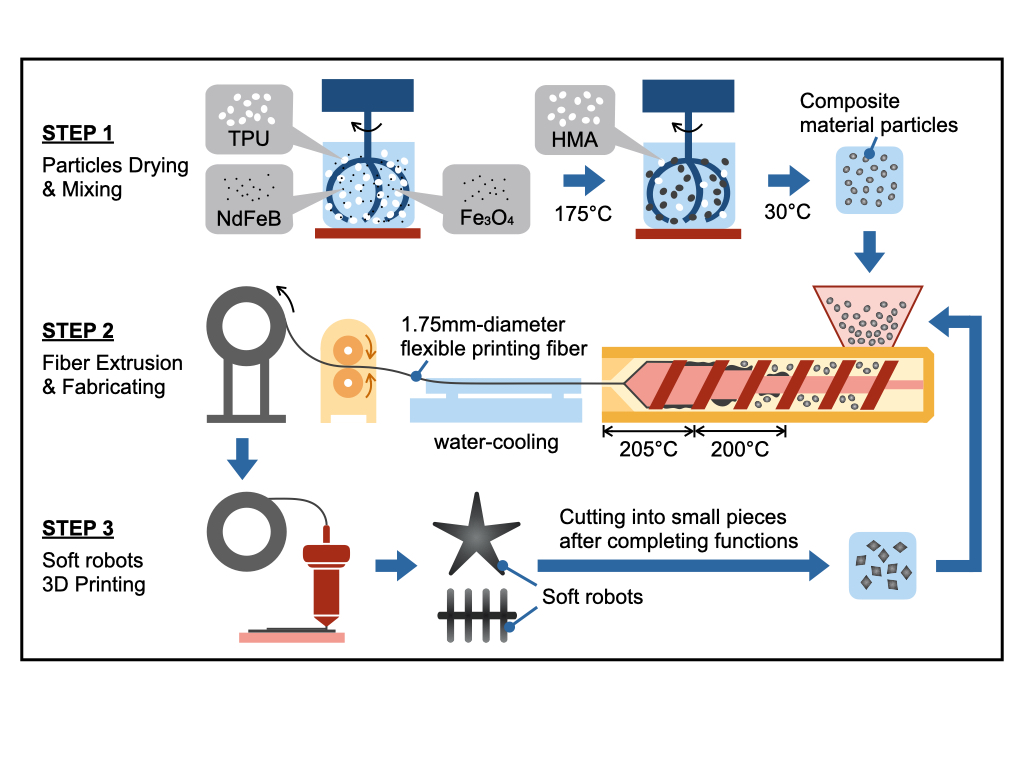
Fig. S1. Fabrication processes of soft fibers and soft robots.** Fabrication processes mainly consist of three steps. In step 1, NdFeB powder and Fe_3_O_4_ powder are added to the dried TPU particles, heated and stirred at 175 °C, and then HMA particles are added and stirred at 30 °C to finally obtain evenly distributed composite material particles. In step 2, the composite material particles are added to the hopper of the single-screw extruder, and the temperature of the mixing section is 200 °C, and the temperature of the extrusion section is 205 °C. The extruded filaments pass through the water cooler and are pulled by the tractor to obtain soft fibers. In step 3, the soft fiber 3D printing soft robots are used to shred into pieces after the soft robots complete the functions, which can be put into the hopper of the extruder for recycling.

**
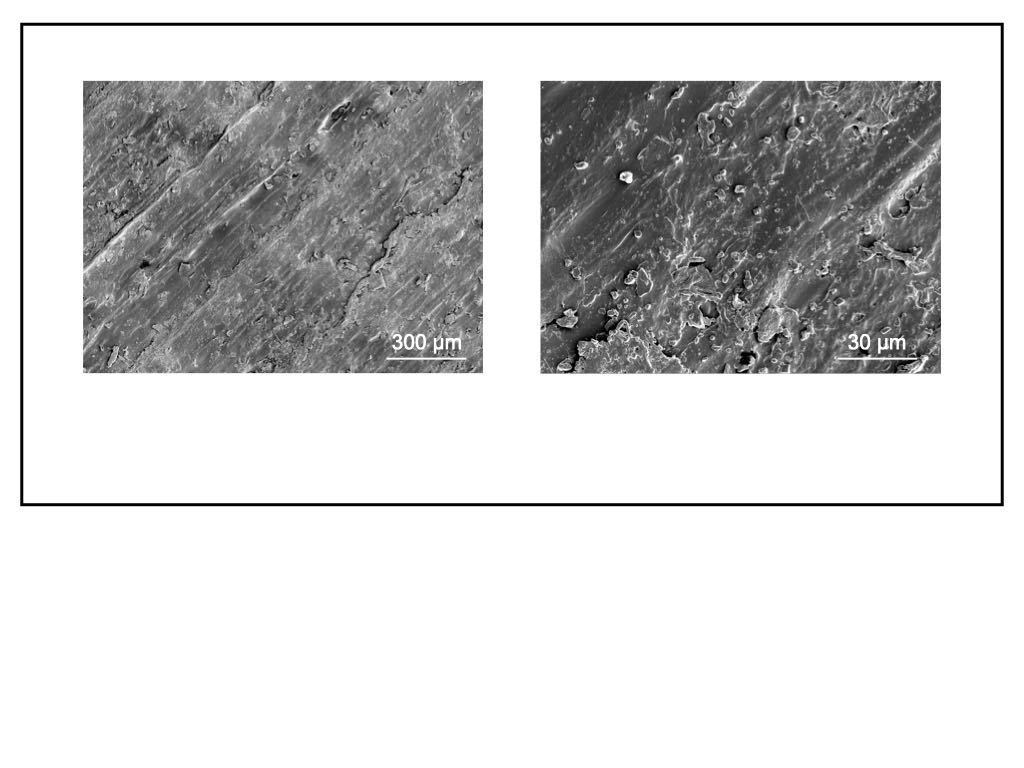
Fig. S2. SEM images of the printed sample surface.** Scanning electron microscopy (SEM) was obtained by Quattro S from Thermo Fisher. The images illustrate NdFeB and Fe_3_O_4_ microparticles can be evenly distributed in TPU-HWA through the full melting process to form an integrated material.


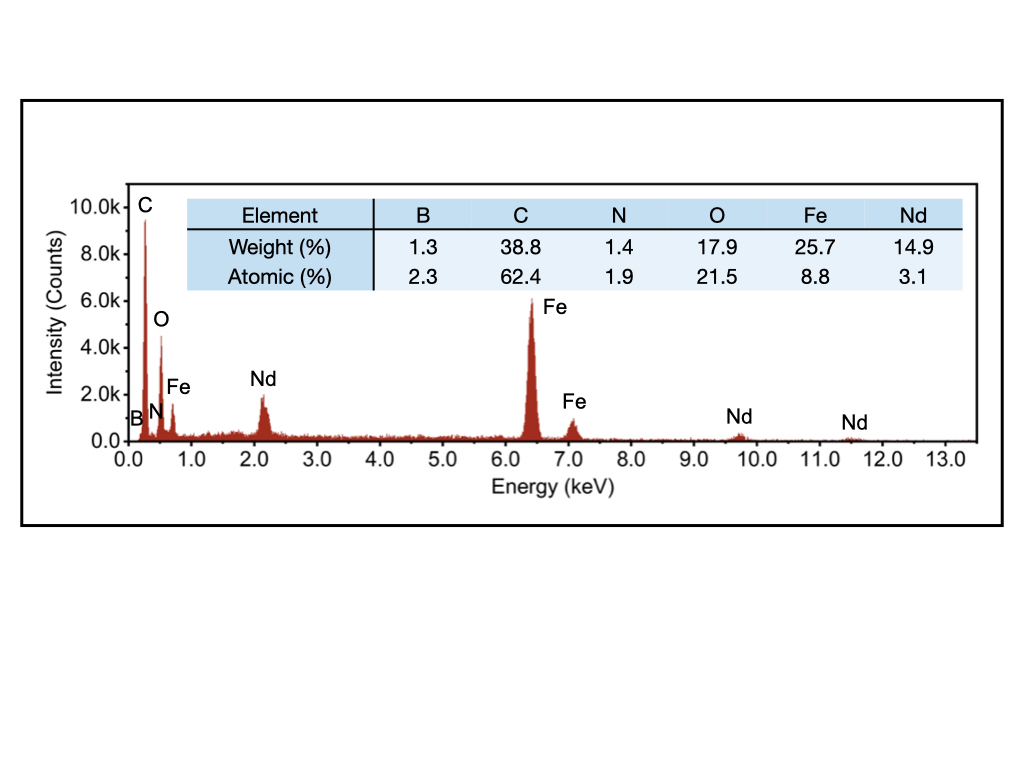
**Fig. S3. Energy dispersive spectroscopy (EDS) image.** Energy dispersive spectroscopy (EDS) was obtained by Octane Elect Plus from EDAX. The image illustrates the presence of B, C, N, O, Fe and Nd elements in soft fibers, and lists the weight ratio and atomic proportion of each element, proving the existence of four materials: TPU, HWA, NdFeB and Fe_3_O_4_.


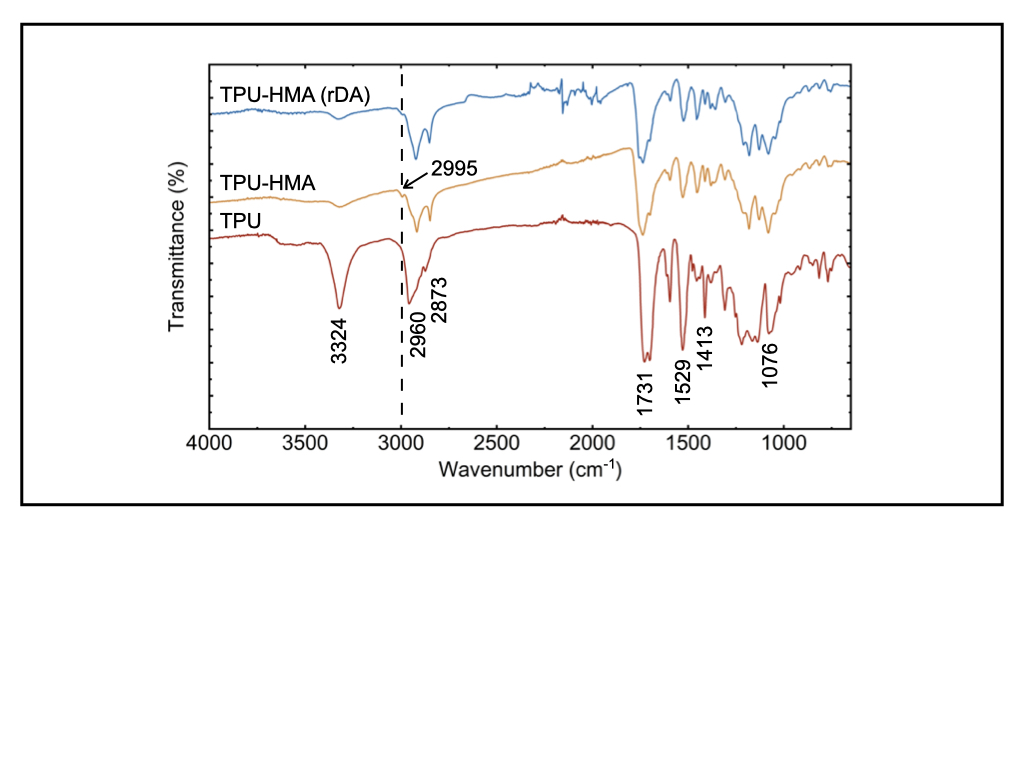
**Fig. S4. Fourier transform infrared spectroscopy (FTIR) - attenuated total reflection mode (ATR) image.** Fourier transform infrared spectroscopy (FTIR) was tested on a Nicolet 380 spectrometer in attenuated total reflectance mode (ATR) with spectral sweep wavenumbers ranging from 600 cm^-1^ to 4000 cm^-1^, 32 scans, and a resolution of 4 cm^-1^. The TPU image was obtained by the TPU sample at room temperature (25 °C), the TPU-HMA image was obtained by the mixed soft fiber sample at room temperature (25 °C), and the TPU-HMA (rDA) image was obtained after the mixed soft fiber sample was heated at 95 °C for 1 hour. The image illustrates the presence of DA bonds, which in turn proves the occurrence of DA reactions.

**
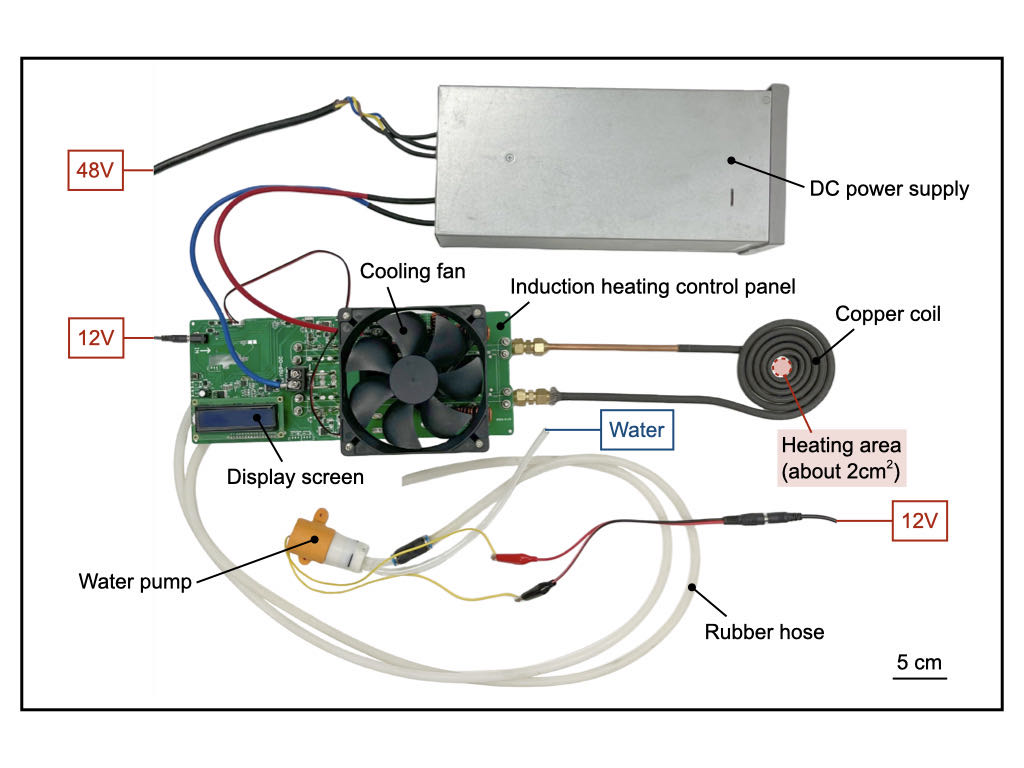
Fig. S5. Diagram of an eddy current heating device.** The eddy current heating device mainly consists of the following five parts: induction heating control panel (used to generate high-frequency alternating current), copper coil (used to generate high-frequency AC heating magnetic field), cooling fan (used to cool the induction heating circuit), DC power supply (used to supply power to the system) and water pump (used to circulate copper coil cooling water).


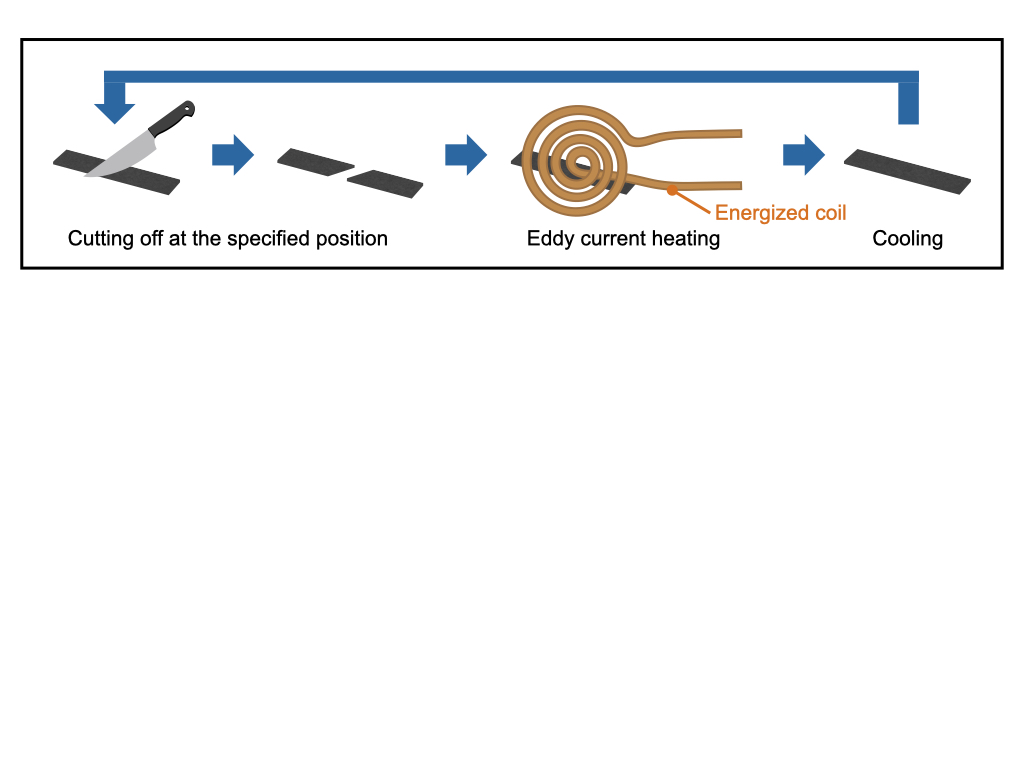
**Fig. S6. Schematic diagram of self-healing performance test.** The middle of the sample is cut obliquely with a knife, the cut is tightly compacted, the eddy current device is connected, and the center of the copper coil is placed directly above the cut for eddy current heating, and then cooled at room temperature. When performing a repeated self-healing experiment, after cooling, the sample is cut again at the first position of the incision, and the self-healing operation procedure is repeated.


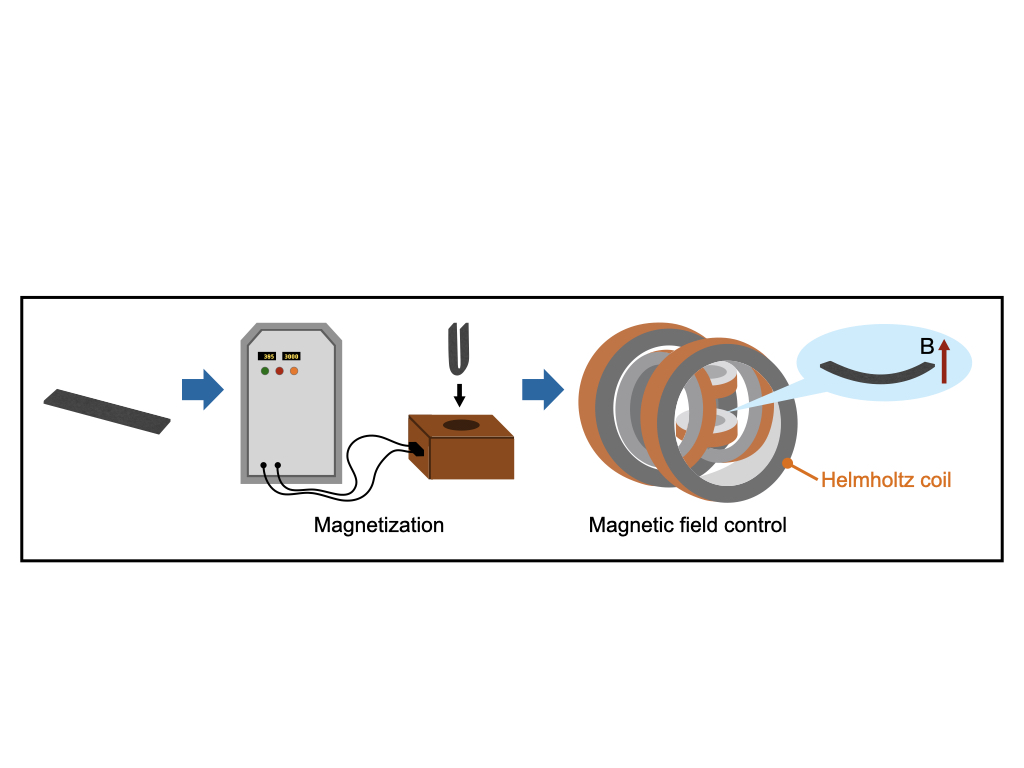


**Fig. S7. Schematic diagram of magnetic actuation performance test.** The sample is fixed into a U-shape with a hard mold, the mold and the soft robot are placed vertically in the box wrapped around the coil, and the magnetizer instantly applies a voltage to generate a pulse field to magnetize the sample. The magnetized sample is placed in the center of the magnetic field control device, and a vertical upward magnetic field is applied to measure the deformation of the sample. In the self-healing magnetron test, the sample should firstly have self-healing test after magnetization.


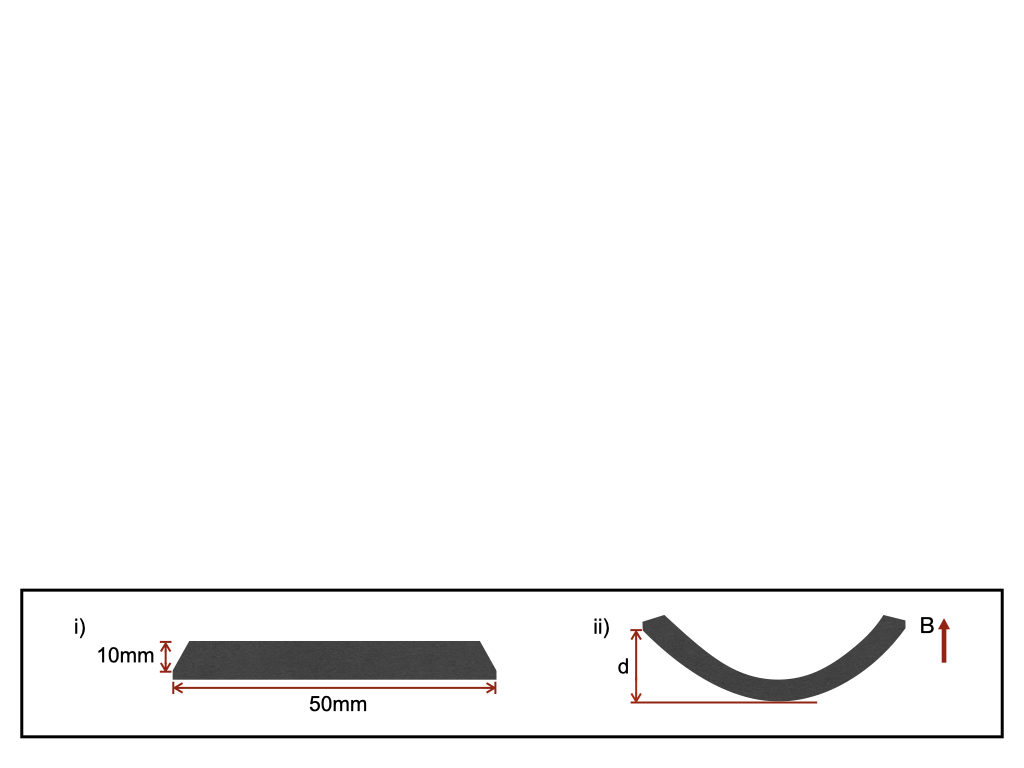
**Fig. S8. Deformation of the magnetic actuation test.** For the magnetic actuation test, some samples are printed (50×10×0.3 mm^3^), as shown in Figure i). The sample is firstly magnetized in the shape shown in Fig. S7. Place the sample in the center of the magnetic field control device, and when the sample is controlled by a vertical upward magnetic field, the two ends of the sample rise, tending to a U-shape. The deformation is defined as the vertical distance between the highest points at both ends and the bottom horizontal plane when the sample is actuated by a vertically upward magnetic field, represented by *d*, as shown in Figure ii).


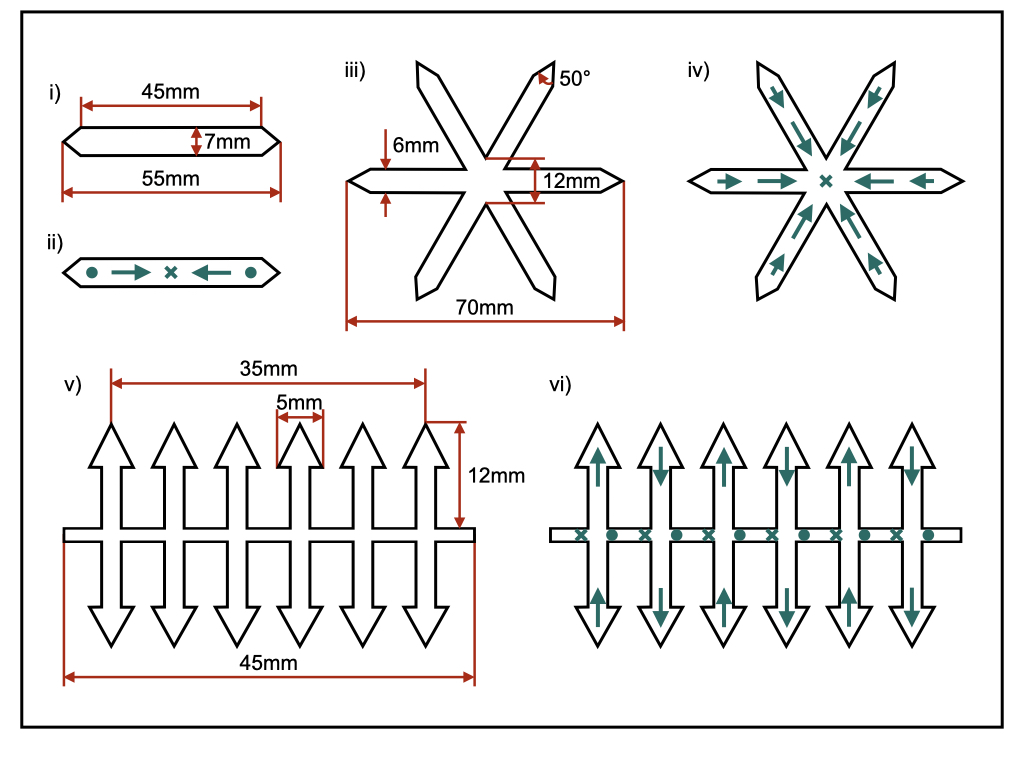


**Fig. S9. Dimensions and magnetic pre-programming status of soft robots.** The dimensions of the soft gripper, soft crawling robot and soft multi-legged robot are shown in Figure i), iii) and v), and the thickness of all these robots is 0.3 mm. The schematic diagram of magnetic pre-programming status of the soft gripper, soft crawling robot and soft multi-legged robot are shown in Figure ii), iv) and vi).

**Movie 1. Fabrication process of soft fibers with an extruder.**

**Movie 2. 3D printing of soft robots using soft fibers.**

**Movie 3. Repeated self-healing process of a soft gripper.**

**Movie 4. Demonstration of a self-healing soft gripper grasping different objects.**

**Movie 5. Transferring process of an object with a self-healing soft gripper.**

**Movie 6. Demonstration of a soft crawling robot transporting an object across a bridge.**

**Movie 7. Self-reconfiguration process of soft crawling robots.**

**Movie 8. The reversible self-reconfiguration process of soft crawling robots.**

**Movie 9. Demonstration of a soft multi-legged robot crawling out of a maze.**

**Movie 10. Demonstration of a soft multi-legged robots traversing complex terrains.**

**Movie 11. Self-amputation escaping process of a soft multi-legged robot.**

**Movie 12. Locomotion of a soft multi-legged robot with and without feet.**
